# Impact on the long-term prognosis of FDG PET/CT in luminal-A and luminal-B breast cancer

**DOI:** 10.1097/MNM.0000000000001500

**Published:** 2021-10-18

**Authors:** Luca Urso, Natale Quartuccio, Matteo Caracciolo, Laura Evangelista, Alessio Schirone, Antonio Frassoldati, Gaspare Arnone, Stefano Panareo, Mirco Bartolomei

**Affiliations:** a Oncological Medical and Specialists Department, Nuclear Medicine Unit, University Hospital of Ferrara, Ferrara; b Nuclear Medicine Unit, A.R.N.A.S. Ospedali Civico, Di Cristina e Benfratelli, Palermo; c Department of Medicine DIMED, Nuclear Medicine Unit, University of Padova, Padova; d Oncological Medical and Specialists Department, Oncology Unit, University Hospital of Ferrara, Ferrara, Italy

**Keywords:** 2- deoxy-2-[18F]fluoro-D-glucose, breast cancer, PET/CT, recurrence, tumor markers

## Abstract

**Purpose:**

The aim of the present study was to explore the prognostic role of 2- deoxy-2-[18F]fluoro-D-glucose PET (FDG PET)/CT in recurrent luminal A and luminal B breast cancer.

**Materials and methods:**

From two institutional databases, we retrospectively retrieved data about breast cancer patients undergoing FDG PET/CT between 2011 and 2018 for the assessment of recurrency. Molecular subtypes of breast cancer were defined based on the expression of estrogen, progesterone, human epidermal growth factor receptor 2 (HER2)-b receptors and proliferation index. Overall survival (OS, intended as the time from PET/CT and the time of death) was registered for each patient, by checking the medical charts. Parametric and survival analyses were computed.

**Results:**

Data of 179 patients were retrieved. Sixty-three patients had luminal A, 88 luminal B and 28 luminal B/He breast cancer. At the time of PET/CT scan, cancer antigen (CA) 15.3 levels was within the normal range in 119 patients, whereas it was increased in 60 patients. FDG PET/CT results were suggestive for disease recurrence in 114 (63.7%) patients. The median time lapse from the FDG PET/CT scan to the last clinical follow-up visit was 51 months (1–192 months). Patients with evidence of a PET/CT scan suggestive for disease recurrence showed a significantly shorter OS (*P* < 0.001) compared to patients with no PET/CT evidence of recurrence, in each subset of luminal breast cancer. Moreover, PET/CT was able to stratify the prognosis of patients independently from the level of tumor marker.

**Conclusion:**

These data suggest that FDG PET/CT may be an attractive prognostic tool in recurrent breast cancer. Our study supports its prognostic role both in luminal A and B-type molecular subtypes, regardless of the CA 15.3 levels.

## Abstract 2

Supplemental Digital Content is available in the text.

## Introduction

Breast cancer is the most diagnosed cancer in women [1] and the second most common malignant tumor worldwide [2]. Approximately 20% of patients will develop metastatic disease, therefore accurate diagnostic imaging modalities are crucial for restaging breast cancer, to select the best treatment approach [2].

Usually, conventional imaging, such as mammography, ultrasonography, computed tomography (CT) and MRI are used. However, the missing of whole-body imaging can affect early detection rate of distant or widespread disease. In this setting, the role of 2- deoxy-2-[18F]fluoro-D-glucose (FDG) PET/CT is still a challenge. It is a recognized useful technique in case of negative or inconclusive conventional imaging results [3–5], mainly if there is an increase in tumor markers [6]. Indeed, elevated serum levels of cancer antigen 15.3 (CA 15.3) often lead to PET/CT scan and are associated with an increased risk of FDG avid metastases [7–11]. However, different breast cancer histopathologic features (i.e. histotype, estrogen receptor-ER expression, tumor grade and proliferation index -Ki67) showed variable FDG avidity [12]. ER-positive breast cancer usually showed a lower FDG avidity as compared to ER-negative ones [13]. Anyhow, within patients affected by hormone-receptor positive breast cancer, FDG PET/CT is potentially a useful tool to discriminate against patients with higher risk of relapse [14]. Nevertheless, the change in biologic characteristics can be found in case of breast cancer recurrence and, therefore, a molecular imaging technique would be useful to guide to a personalized treatment approach. Furthermore, detecting FDG-positive breast cancer metastasis during follow-up is a recognized negative prognostic factor [6,15,16].

The aim of the present study was to explore the prognostic role of FDG PET/CT in recurrent luminal A and luminal B breast cancer.

## Materials and methods

### Patient recruitment

We retrospectively investigated the databases of two Italian hospitals involving breast cancer patients who underwent FDG PET/CT scan for the assessment of suspected and or without diagnosed recurrency, between January 2011 and January 2018. Inclusion criteria were: (1) a biopsy-proven breast cancer; (2) execution of FDG PET/CT scan for the assessment of recurrent disease; (3) absence of other concomitant malignancies and (4) the availability of follow-up information. Only patients who underwent FDG PET/CT in the postoperative setting were included. According to both institutional policies, all patients gave their informed consent to undergo FDG PET/CT scanning, and to any subsequent, anonymized analysis of their data.

### 2- deoxy-2-[18F]fluoro-D-glucose PET/computed tomography acquisition protocol

All patients were required to fast at least for 6 h and maintain adequate hydration before the scan. Diabetic patients had blood glucose measured before FDG delivery. Those patients with fasting glucose above 200 mg/dl were postponed until proper therapy was established. The FDG injection (1 mCi/10Kg) was performed 60 min before image acquisitions. Two dedicated PET/CT systems (Siemens Biograph mCT Flow and Siemens Biograph 16S, respectively at Ferrara and Padova hospital) were used. Data acquired with the CT scan were used for attenuation correction and fused with PET images. PET/CT data were reconstructed using a dedicated commercial workstation along axial, coronal and sagittal.

### Imaging analysis and collection of data

A resident doctor and a referring nuclear medicine physician with at least 10-year experience visually evaluated the evidence of disease recurrence at PET/CT images, by using a dedicated workstation. Discordant readings by the two observers were evaluated by a third blinded nuclear medicine physician. The site of recurrence, defined as the presence of an abnormal FDG uptake outside from the physiologic distribution and compatible with the widespread breast cancer, was noted in a dedicated spreadsheet. FDG uptake in the site of the primary tumor (T) or in local and distant lymph nodes (L) or in the distant organs (i.e. liver, bone, lung and others, M) was appropriately described in the database. The accuracy of FDG PET/CT was not verified by pathology or imaging/clinical follow-up given that the aim of the present study was primarily about its prognostic value.

### Clinical data and CA 15.3

For each patient, demographic and clinical data were appropriately recovered. Four main intrinsic or molecular subtypes of breast cancer were defined based on the luminal classification: (1) luminal A [positive ER and/or progesterone-receptor-PR, negative human epidermal growth factor receptor 2 (HER2) and levels of the protein Ki-67 lower than 14%], (2) luminal B or luminal B/He (positive ER and/or PR, and either negative HER2 or positive HER2 with levels of Ki67 higher than 14%) and (3) triple-negative/basal-like (negative ER and PR and negative HER2) and HER2-enriched (ER and PR negative and HER2 positive) [17]. Fluorescence in situ hybridization (FISH) was used to discriminate between positive and negative HER2 breast cancer. Moreover, if available, CA 15.3 values within 3–4 months before PET/CT scan were also extracted. CA 15.3 levels were considered normal in a range between 0 and 32 UI/ml. Follow-up data were collected by checking the medical chart or by a telephone interview.

### Statistical analysis

The distribution of data was tested by using the normality test (Shapiro-wilk). Continuous variables were expressed as mean (SD, DS) and categorical variables as number (percentage).

Differences in proportions of frequency for stage, histotype and grade were assessed among the three luminal subtypes by means of the chi-square test at a 0.05 level of significance.

Overall survival (OS) was defined as the time interval between restaging PET/CT and the date of cancer-related death. Kaplan–Meier analysis and log-rank tests were used for the survival analysis. The significance of the difference in survival between different groups was set at a threshold of *P* < 0.05. The prognostic impact on OS of positive PET/CT results was evaluated in the whole population and in the different molecular subtypes assessing the difference in OS based on the different locations of recurrence. Finally, we compared the OS of patients with normal CA 15.3 levels and positive PET/CT findings with that of patients with normal CA 15.3 values and no evidence of recurrence at the restaging PET/CT scan.

All statistics were performed in IBM SPSS version 22 (IBM SPSS statistics for Windows; IBM Corp., Armonk, New York, USA) and MedCalc Statistical Software version 19.1.3 (MedCalc Software, Ostend, Belgium; https://www.medcalc.org; 2020).

## Results

Data of 179 patients were retrieved. Sixty-three patients had luminal A, 88 luminal B and 28 luminal B/He breast cancer. Patient demographic and clinical information are displayed in Table 1. The distribution of clinical data was similar across the groups, although the number of patients with a grade III breast cancer was slightly higher in luminal B and luminal B/He than that in luminal A.

**Table 1:** Demographic and clinical information on patient population

Variables	All patients (*n* = 179)	Luminal category		
		A (*n* = 63)	B (*n* = 88)	B/He (*n* = 28)
Median age (range), years	61 (32–90)	62 (32–82)	61 (34–90)	60 (36–72)
Histotype, *n* (%)				
Ductal	54 (30.2%)	9 (14.3%)	33 (37.5%)	12 (42.9%)
Lobular	95 (53%)	38 (60.3%)	41 (46.6%)	16 (57.1%)
Ductal and lobular	9 (5%)	5 (7.9%)	4 (4.6%)	–
Papillar	19 (10.6%)	10 (15.9%)	9 (10.2%)	–
Glycogenic cells	2 (1.1%)	1 (1.6%)	1 (1.1%)	–
Grade, *n* (%)				
I	48 (26.8%)	22 (34.9%)	21 (23.9%)	5 (17.8%)
II	63 (35.2%)	19 (30.2%)	33 (37.5%)	11 (39.3%)
III	25 (13.9%)	1 (1.6%)	16 (18.2%)	8 (28.6%)
Unknown	43 (24%)	21 (33.3%)	18 (20.4%)	4 (14.3%)
TNM stage, *n* (%)				
I	62 (34.6%)	21 (33.3%)	33 (37.5%)	8 (28.6%)
II	56 (31.3%)	21 (33.3%)	26 (29.5%)	9 (32.1%)
III	31 (17.3%)	12 (19.1%)	14 (15.9%)	5 (17.9%)
IV	30 (16.8%)	9 (14.3%)	15 (17.1%)	6 (21.4%)

TNM, tumor node metastases.

At the time of PET/CT scan, CA 15.3 level was within the normal range in 119 patients, whereas it was increased in 60 patients. FDG PET/CT results were suggestive for disease recurrence in 114 (63.7%) patients. Three patients presented only local breast recurrence, 27 showed lymph node recurrence without distant metastases, and 84 had distant metastatic lesions (Table 2). The distribution of recurrence at FDG PET/CT was quite similar in all the groups. Conversely, CA 15.3 was more often abnormal in patients with luminal B breast cancer as compared to those with luminal A or luminal B-He breast cancer.

**Table 2:** Biochemical and PET/CT findings in the luminal category

Variables	All patients (*n* = 179)	Luminal category		
		A (*n* = 63)	B (*n* = 88)	B/He (*n* = 28)
PET/CT, *n* (%)				
Negative	65 (36.3%)	25 (39.7%)	32 (36.4%)	8 (28.6%)
Positive	114 (63.7%)	38 (60.3%)	56 (63.6%)	20 (71.4%)
Local recurrence at PET/CT, *n* (%)				
No	176 (98.3%)	62 (98.4%)	87 (98.9%)	27 (99.4%)
Yes	3 (1.7%)	1 (1.6%)	1 (1.1%)	1 (0.6%)
Lymph node recurrence at PET/CT, *n* (%)				
No	152 (84.9%)	56 (88.9%)	73 (83%)	23 (82.4%)
Yes	27 (15.1%)	7 (11.1%)	15 (17%)	5 (17.6%)
Distant recurrence at PET/CT, *n* (%)				
No	95 (53%)	33 (52.4%)	48 (54.5%)	14 (50%)
Yes	84 (47%)	30 (47.6%)	40 (45.5%)	14 (50%)
CA 15.3 level, *n* (%)				
Normal	119 (66.5%)	46 (73%)	51 (57.9%)	22 (78.6%)
Abnormal	60 (33.5%)	17 (27%)	37 (42.1%)	6 (21.4%)

**Fig. 1:**
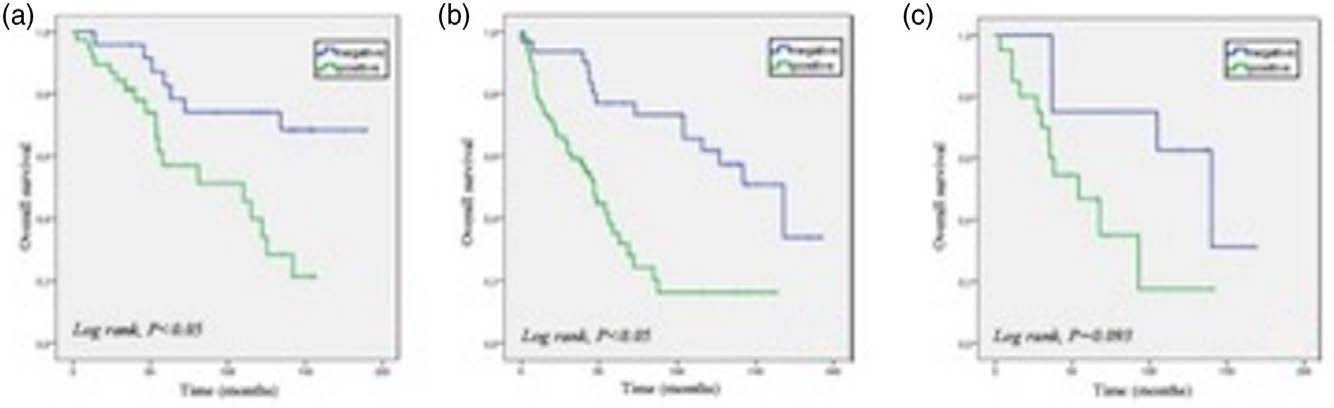
Kaplan–Meier analysis for PET/CT results in patients with luminal A (a), luminal B (b) and luminal B-He breast cancer (c).

The median time lapse from the FDG PET/CT scan to the last clinical follow-up visit was 51 months (1–192 months). In the whole study group, patients with evidence of a PET/CT scan suggestive for disease recurrence at any site showed a significantly shorter OS (*P* < 0.001) compared to patients with no PET/CT evidence of recurrence (Supplementary Figure 1s, Supplemental digital content 1, http://links.lww.com/NMC/A206). No significant difference was found comparing the OS of patients with N-recurrence (*n* = 27) from that of patients with M-recurrence (*n* = 84). Subjects with luminal A subtype demonstrated a significantly longer OS than patients with B or B/He subtype (overall comparison: *P* = 0.033). The OS of patients with luminal A subtype and negative PET/CT scan (*n* = 25) was significantly longer than the OS of subjects with luminal A subtype and a positive PET/CT scan (*n* = 38; *P* = 0.04) (Fig. 1a). Also, luminal B patients with a negative PET/CT scan (*n* = 32) showed a better outcome than luminal B patients with a positive PET/CT scan (*n* = 56; Fig. 1b). In patients with the luminal B/He subtype, no difference in survival was observed between patients with positive (*n* = 20) and negative (*n* = 8) PET/CT (*P* = 0.093; Fig. 1c). In Supplementary Figure 2, Supplemental digital content 2, http://links.lww.com/NMC/A207 are reported the Kaplan–Meier curves based on the luminal category and in accordance with FDG PET/CT results.

### Prognostic value of FDG PET/CT in patients with normal CA 15.3 levels

There were 119 cases of normal CA 15.3 levels among the patients. Sixty-nine out of 119 cases presented positive FDG PET findings. Areas of increased FDG uptake were in the breast in 3 cases, in the lymph nodes in 15 cases and in other sites (distant metastases in 51 scans). The frequency of histotype for these 69 cases was similar for ductal and lobular histotypes (47.8% vs. 42%). The frequency of distribution was similar also for the luminal categories. No difference was found between luminal A and B, for CA 15.3 levels and PET/CT findings (Table 3).

**Table 3:** Distribution of PET findings and CA15.3 levels based on the luminal A or B breast cancer

Variable	Luminal A (*n* = 63)	Luminal B (*n* = 88)	*P value*
PET result			
Negative	25 (39%)	32 (36%)	0.678
Positive	38 (61%)	56 (64%)	
Ca 15.3 level			
Normal (<32 UI/mL)	46 (73%)	51 (58%)	0.057
Abnormal (>32 UI/mL)	17 (27%)	37 (42%)	

Abnormal CA 15.3 and negative FDG PET/CT were present in 15 patients (mean age: 55 ± 10) who showed an OS of 88.27 ± 55.31 months. Histotype was lobular in 10/15 cases, mixed ductal-lobular in one patient and papillar in four cases. Eight patients had luminal A, six luminal B and one B/He breast cancer. No significant difference in titer of CA 15.3 was detected when comparing positive vs. negative HER2-breast cancer (mean titer: 31.9 vs. 64.2 UI/mL, respectively).

**Fig. 2:**
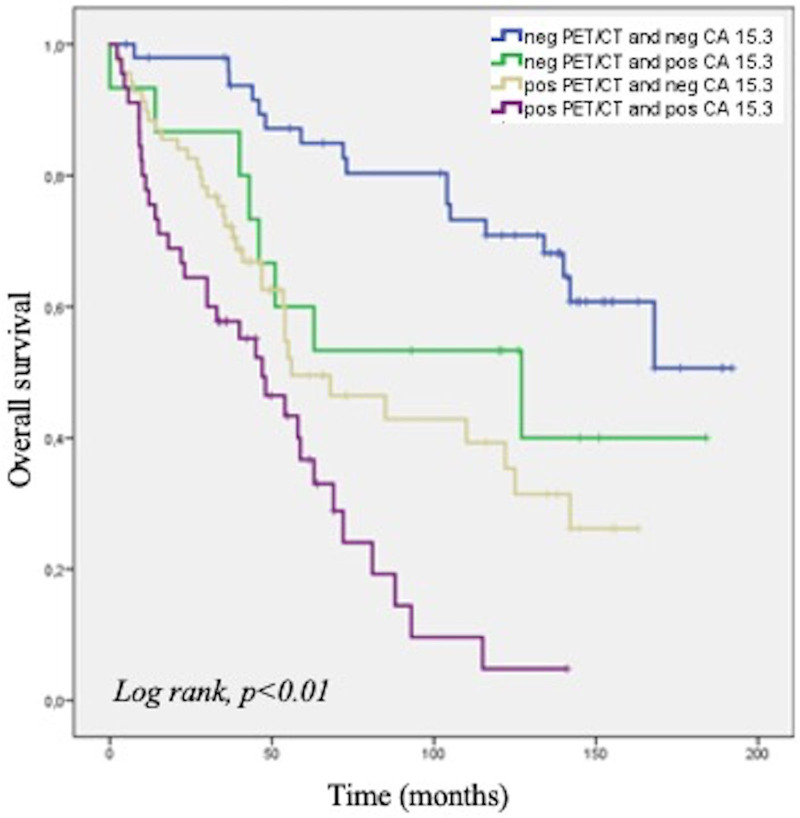
Kaplan–Meier analysis for PET/CT and CA15.3 results in all patient populations.

Figure 2 shows the OS curves in all patient’s population, based on CA 15.3 levels and PET/CT results. As clearly illustrated, a positive PET/CT was correlated with a worse prognosis, independently from CA 15.3 levels.

**Fig. 3:**
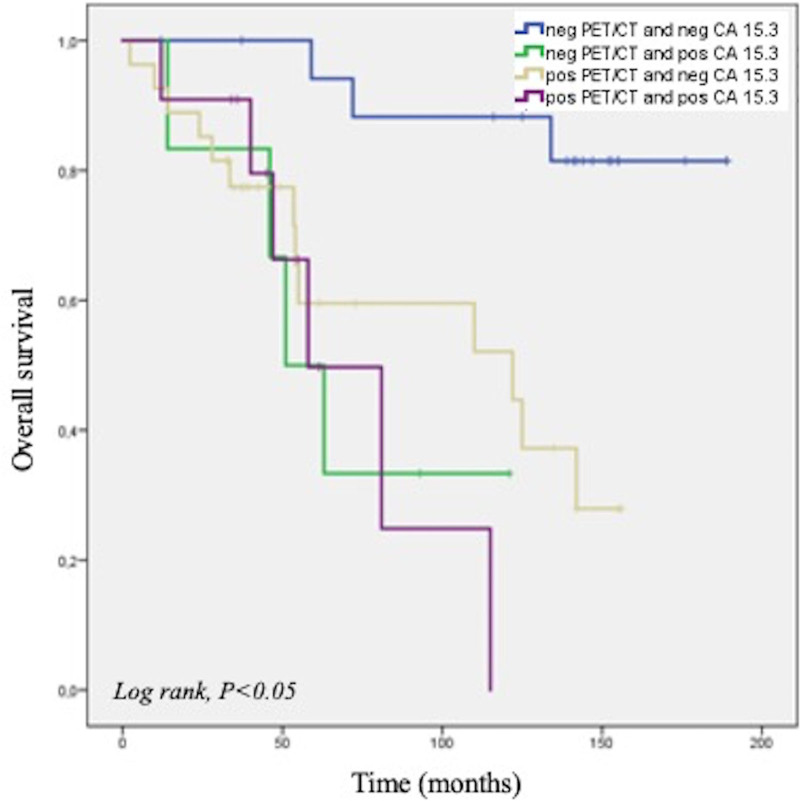
Kaplan–Meier analysis for PET/CT and CA15.3 results in luminal A breast cancer patients.

The OS of women with luminal A subtype was significantly shorter when CA 15.3 levels were increased (*n* = 17) at restaging measurement (*P* = 0.007). In the 46 luminal A subtype patients with a negative CA 15.3 measurement, PET/CT findings further stratified patient survival (*P* = 0.002; Fig. 3). In detail, the 19 patients with normal CA 15.3 level and no evidence of recurrence at PET/CT showed a longer OS (95% CI, 151.13–190.33 months) compared to the 27 patients with normal CA 15.3 and a PET/CT suggestive for disease recurrence (95% CI, 73.88–120.65 months) (Fig. 3).

**Fig. 4:**
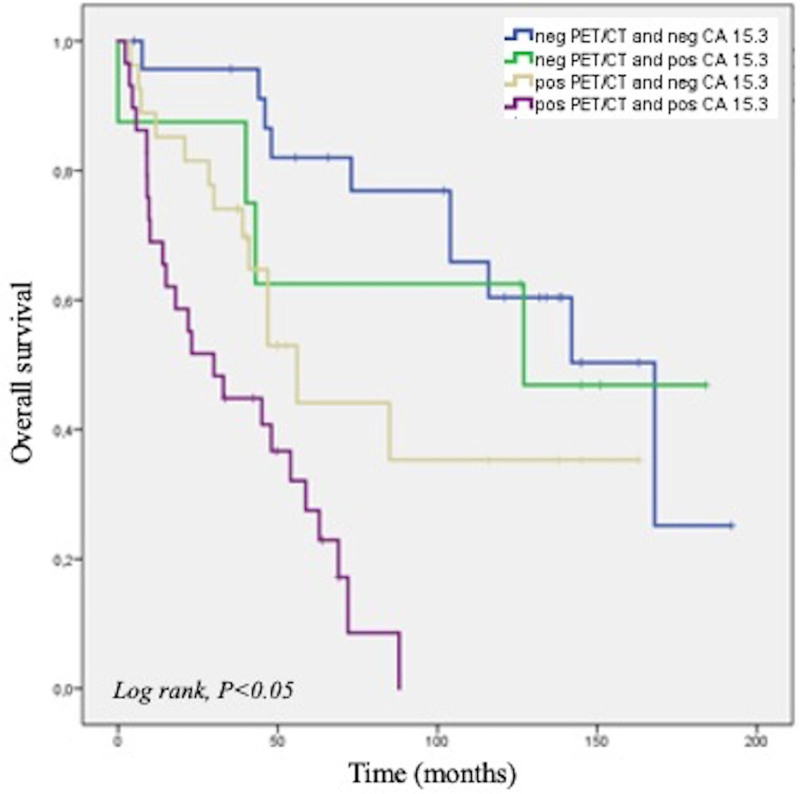
Kaplan–Meier analysis for PET/CT and CA15.3 results in luminal B breast cancer patients.

The OS of women with luminal B subtype was significantly shorter when CA 15.3 levels were increased (*n* = 37) at restaging measurement (*P* = 0.01). In the 51 luminal B subtype patients with a negative CA 15.3 measurement, PET/CT findings further stratified patient survival (*P* = 0.037; Fig. 4). The 24 patients with normal CA 15.3 level and no evidence of recurrence at PET/CT showed a longer OS (95% CI, 107.21–158.83 months) compared to the 27 patients with normal CA 15.3 and a PET/CT suggestive for disease recurrence (95% CI, 54.44–112.12 months) (Fig. 4). For luminal B/He patients, no significant difference in survival was detected between subjects with normal and increased CA 15.3 level.

## Discussion

FDG PET/CT still stands as a second-line imaging option in restaging patients with breast cancer, despite its widely documented clinical utility [18]. Indeed, FDG PET/CT detection rate of metastatic lesions is high either in presence of clinical suspicion of recurrence or in presence of increasing tumor markers over time, with a pooled sensitivity of 90 and 87.8%, respectively, according to two recent meta-analyses [6,19]. In presence of combined abnormal CA 15.3 levels and radiologic suspicion of recurrence, even higher sensitivity (92.7%) has been documented [10]. Furthermore, PET/CT appears superior to most conventional imaging techniques (i.e. CT, bone scintigraphy and ultrasonography) in detecting recurrence, although not outperforming MRI [20,21]. Moreover, PET/CT has a relevant impact on patient management at restaging, due to its high negative predictive value compared to conventional imaging [3]. A recent meta-analysis by Pak *et al*., [22] showed that the pooled rate of management change for FDG PET/CT in the subgroup of patients with elevated tumor markers was 52.3%. In the present study, we found that FDG PET/CT results were suggestive for disease recurrence in 63.7% of selected patients, being positive for distant metastatic lesions in 74% of them, thus prompting an appropriate staging of disease.

The prognostic value of FDG PET/CT in breast cancer patients at restaging has been proved in few studies either in female or male subjects [5,16]. In our study, we collected a large bicentric patient population divided in groups based on the molecular subtype. We demonstrated that the presence of pathologic findings at restaging PET/CT is a negative prognostic factor either in patients with luminal A or luminal B-B/He+ patients. In keeping with our study, also Aogi *et al*., [15] demonstrated the prognostic relevance of FDG PET/CT in a large series of 262 stage I-III patients with luminal A or B type. However, Aogi *et al*., [15] selected a group of patients with breast cancer, in the preoperative setting.

As largely reported, breast cancer is a heterogeneous disease, and among ER-positive diseases, some of them have aggressive behavior [23,24]. The uptake of FDG detected by PET/CT can detect this aggressiveness, and thus guide to appropriate treatment approaches, also in the restaging phase. Indeed, we found that the OS was significantly worse in patients with a luminal A/B BC with a positive PET/CT as compared to those with a negative scan. Therefore, the opportunity to test *in vivo* the evidence of aggressive recurrence can lead to a tailored therapy, to improve the long-term outcome.

Triple-negative breast tumors are usually FDG-avid [25], whereas the FDG uptake in HER2 positive breast cancer is widely variable [26,27]. PET/CT has been tested both in patients with triple-negative and HER2 positive breast cancer, mainly in determining the prognostic meaning of change in metabolism, before and after neoadjuvant therapy and prognosis. Akimoto *et al*., enrolled 130 patients with HER2 positive (53% of cases) and triple-negative (40% of cases) who underwent FDG PET/CT before and after neoadjuvant chemotherapy [28]. The authors found that the value of standardized uptake value (SUV)_max_ in the primary lesion, after neoadjuvant chemotherapy was a significant prognostic factor, being associated with recurrence-free survival (patients with high post-SUV_max_ have a poor prognosis). However, to our knowledge, no specific data are available about the prognostic role of FDG PET/CT in recurrent triple-negative or purely HER2 positive breast cancer, that is beyond the aim of the present article.

In the present study, we found a slight increase in CA 15.3 levels in luminal B patients than luminal A (42 vs. 27%, respectively; see Table 2), independently from the results of PET/CT. A potential explanation for this disparity can be found in the different proliferation index of the histotype. Luminal A and luminal B, based on the St. Gallen classification, are defined in the presence of a proliferation index < and > 14%, respectively. A high proliferative tumor can produce more CA 15.3; moreover, a luminal disease produces more CA 15.3 than a nonluminal disease [29]. As reported by Geng *et al*., [29], less differentiated subtypes of breast cancer lack certain circulating antigens. Moreover, the elevation of tumor markers depends on the site of recurrence (i.e. liver metastases and pleural effusion seems correlated with high CA 15.3 levels, [30]). In the present study, patients with luminal A and luminal B showed a positive PET/CT in lymph node and in distant metastases at PET/CT, respectively in 11.1 and 47.6% vs. 17 and 45.5% cases. Therefore, the widespread of disease was slightly higher in luminal B than luminal A.

The novel finding of our study is the possibility to further stratify OS regardless of the level of CA 15.3. At restaging, positive PET/CT findings, despite CA 15.3 within normal range, were associated with worse long-term survival in our patient population. Until now, no study has ever investigated the combined prognostic role of CA 15.3 and FDG PET/CT findings in patients with luminal A or luminal B breast cancer. Therefore, during follow-up, although negative CA 15.3 levels, PET/CT would be useful for the stratification of this subset of patients.

The current international guidelines did not mention FDG PET/CT as a first- or second-line imaging modality in patients with breast cancer, during follow-up. It should be used only in case of doubtful findings at conventional imaging or in case of an increase in tumor markers, but with a negative conventional imaging result. However, as emerged from the present study, FDG PET/CT should be performed, independently of the level of CA 15.3, because it has a strong prognostic impact.

In addition to FDG, another radiopharmaceutic, 18F-fluoroestradiol (FES) may emerge in the future as a complementary imaging tool characterizing noninvasively additional tumor features, related to the expression of ER [31]. The combination of FDG and FES, in terms of metabolic and receptor information, may increase the ability to delineate the heterogeneity of breast cancer recurrence, thus guiding to hormonal or systemic treatments or mixing of them. The choice between FDG or FES, as a radiopharmaceutic agent, depends on the histopathologic characteristics of the tumor. Patients with luminal A or luminal B breast cancer would have benefited from both the agents, whereas patients with ER-/PR-/HER2 positive or triple-negative tumors would receive only FDG PET/CT. Other radiopharmaceutics that have been proposed as alternatives to FDG are fluciclovine [32] and fluorothymidine [33]. Nevertheless, experience with these radiopharmaceutics is currently limited to few clinical studies involving patients with breast cancer; therefore, preventing drawing compelling conclusions.

A limitation of our study concerns the retrospective study design, in keeping with most of the available literature on the utility of FDG PET imaging to predict prognosis at restaging. Another source of error may be related to the wide interval of patient age at first diagnosis. Although the collection of data was bicentric and interpretation of PET/CT images was not centralized, we believe that no interpretation bias has occurred due to long-lasting experience of the nuclear medicine physicians involved in the study. In consideration of the long-standing expertise of the nuclear medicine physicians involved in the study, we believe that no centralization of imaging interpretation was necessary. Another potential limitation is the minimum time of follow-up in some patients. Eight patients had a follow-up shorter than 6 months, and seven out of these eight patients died. PET was positive in six out of the seven patients who died and negative in the remainder case who was still alive at the end of the follow-up. CA 15.3 level in these eight patients was quite variable, ranging from 22 to 729 UI/mL). Patients had luminal B breast cancer in six cases, luminal A breast cancer in one case and luminal B/HE breast cancer in one case. However, due to the limited number of patients with a short follow-up, we decided to include them in the final analysis.

### Conclusion

At restaging, FDG PET/CT in luminal A and luminal B may provide powerful prognostic information; furthermore, it may be used to further stratify patients with CA 15.3 within normal range. Prospective studies are warranted to corroborate these retrospective findings.

## Acknowledgements

All procedures performed in studies involving human participants were in accordance with the ethical standards of the institutional and/or national research committee and with the 1964 Helsinki declaration and its later amendments or comparable ethical standards. For this type of study (a retrospective trial) formal consent is not required.

Conception or design: N.Q., L.E. and S.P. Acquisition, analysis and interpretation if the data: L.U., M.C., L.V., L.E. and N.Q. Drafting or revising for important intellectual content: L.U., M.C., L.E. and N.Q. Final approval of the version: all authors.

## Conflicts of interest

There are no conflicts of interest.

## Supplementary Material
