# Supplementary figures and images for: Impact on the long-term prognosis of FDG PET/CT in luminal-A and luminal-B breast cancer
Source: Nucl Med Commun. 2021 Oct 18;43(2):212–9. doi: 10.1097/MNM.0000000000001500 (PMC10876173; doi:10.1097/MNM.0000000000001500)

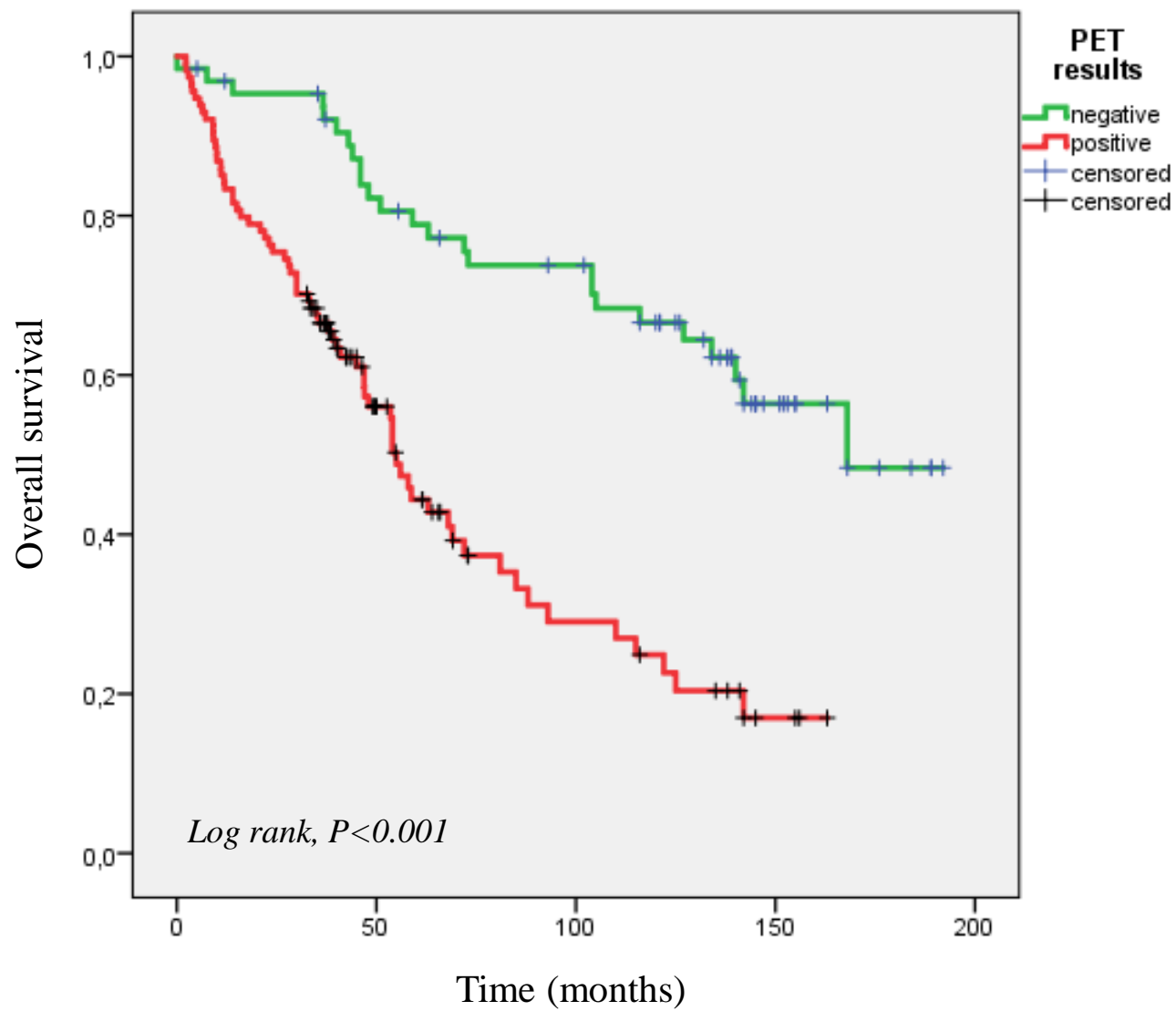

Supplement: Supplementary file 1 [file nmc-43-212-s001.pdf]

Negative FDG PET/CT

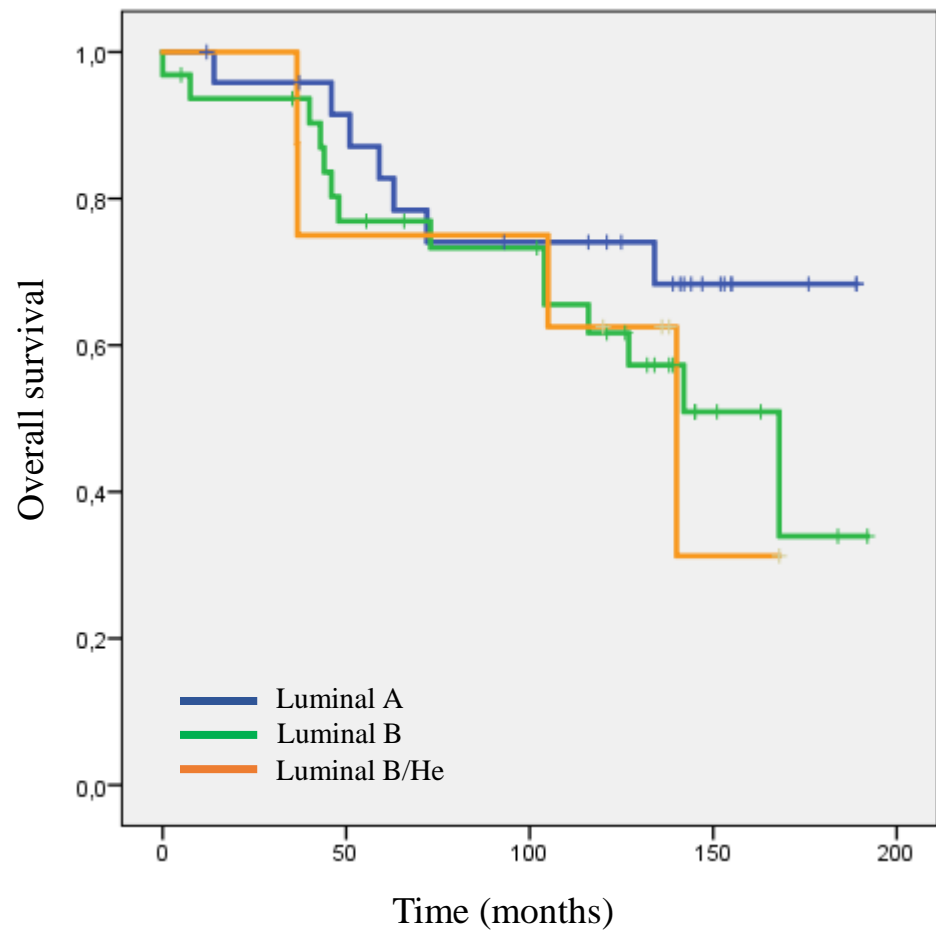

Positive FDG PET/CT

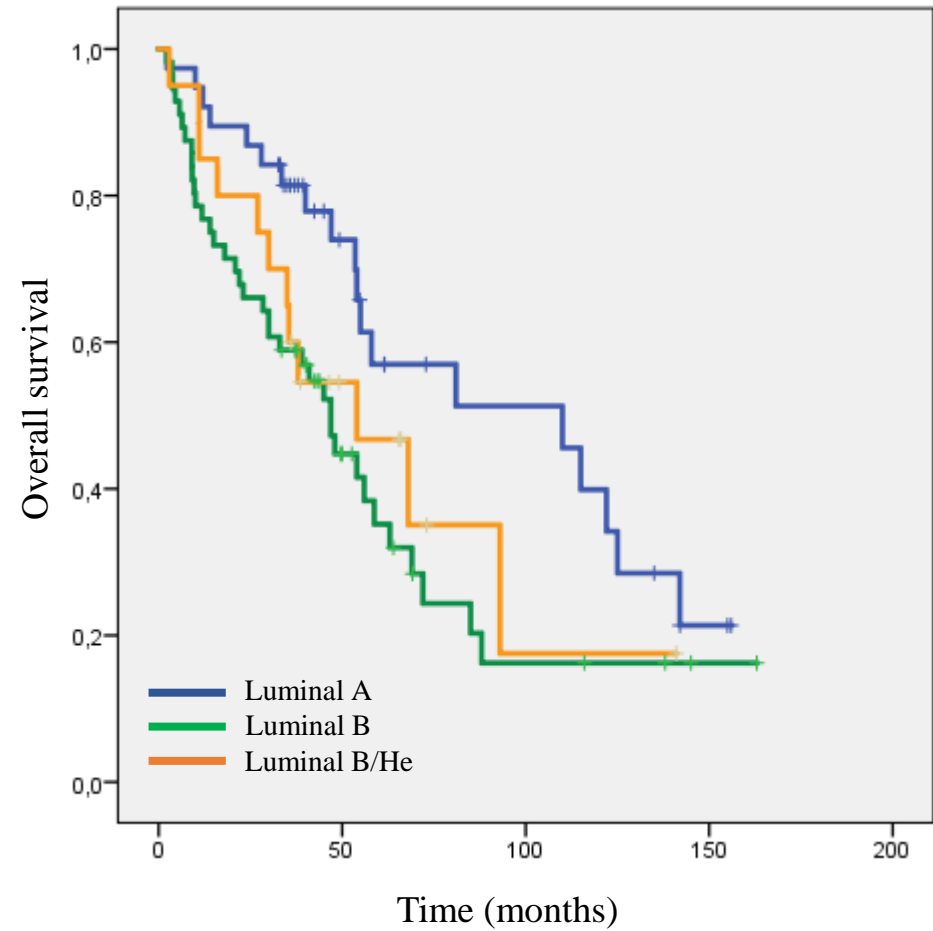

Supplement: Supplementary file 2 [file nmc-43-212-s002.pdf]
